# Local clinical practice patterns in urolithiasis guidelines: a critical evaluation from Turkey

**DOI:** 10.1007/s00345-025-05490-y

**Published:** 2025-02-03

**Authors:** Kemal Sarica, Rasim Güzel, Zeki Bayraktar, Salih Yildirim, Hikmet Yasar, Göksu Sarica, Cahit Sahın

**Affiliations:** 1 Department of Urology, Sancaktepe Research and Training Hospital, Health Sciences University, Istanbul, Turkey; 2 Department of Urology, Biruni University Medical School, Istanbul, Turkey; 3 Urology Clinic, Kavacık Medistate Hospital, Istanbul, Turkey; 4 Medical intern, University Medical School, Istanbul, Turkey

**Keywords:** Guidelines, Clinical practice patterns, Urolithiasis, Survey, Local

## Abstract

**Purpose:**

This study aimed to evaluate the current clinical practice patterns regarding the utilization of “Urolithiasis Guidelines” in Turkey and to identify critical factors influencing their application by urologists.

**Methods:**

The study targeted practicing urologists in Turkey, primarily those involved in the management of urolithiasis, to assess their perspectives and experiences regarding the clinical application of established guidelines. A total of 415 urology specialists were invited to participate in a survey-based study conducted via Google Forms. Participation was voluntary, and 65.08% of the invited urologists completed the survey.

**Results:**

Among the respondents, 84.7% reported utilizing the available guidelines in their routine clinical practice, with varying frequencies of reference. The primary motivations for guideline use were the prevention of potential complications and the avoidance of legal risks, as indicated by 90.5% of respondents. While 56.9% of participants adhered to the guidelines as a clinically standardized practice, 41.6% reported applying the recommendations on a case-by-case basis. Notably, 41.0% of respondents emphasized the need for locally adapted versions of guideline texts. Additionally, nearly half of the participants reported receiving no formal education or training on the significance, content, and practical application of these guidelines. Furthermore, 12.7% expressed skepticism about the evidence-based foundation of the guidelines, questioning whether the recommendations were derived from rigorously conducted studies.

**Conclusion:**

The available urolithiasis guidelines are recognized as valuable resources offering key recommendations for the effective and safe management of urolithiasis. However, findings from this survey highlight significant variability in clinical practice patterns due to local conditions and the individual experience and attitudes of practicing urologists. The application of guideline recommendations is further influenced by perceptions regarding their development, content, and practicality. Insights gathered from this study may contribute to improving the preparation, dissemination, and implementation of urolithiasis guidelines, particularly in adapting them to local clinical settings.

**Supplementary Information:**

The online version contains supplementary material available at 10.1007/s00345-025-05490-y.

## Introduction

The primary objective of clinical guidelines is to provide actionable recommendations for practicing physicians, predominantly derived from the outcomes of rigorously conducted research studies. These guidelines hold significant importance in the medical field, offering a foundation for individualized and rational decision-making aimed at enhancing healthcare quality [1–3]. 

Achieving optimal clinical outcomes with minimal or no complications is an anticipated result when management approaches are aligned with guideline recommendations. However, it is well established that reliable and widely accepted recommendations can only be formulated through a robust methodological approach. A systematic review of evidence-based data from well-designed clinical and research trials constitutes the cornerstone of guideline development.

Despite the strength of these guidelines, their feasibility and clinical application depend on several critical factors, including patient acceptance, the availability of requisite infrastructure, and, most importantly, adequate expertise in the specific management modalities being implemented. Beyond their role for practicing physicians, these guidelines serve as a crucial reference for patients, policymakers, insurance providers, educational programs, and forensic medicine. Consequently, the reliability, quality, and validity of these guidelines rely on scientifically sound data acquisition, the quality of included trials, the methodologies employed in critical evaluations, and the alignment between derived data and the formalization of evidence levels for users [4–6].

While the theoretical benefits of such guidelines are widely recognized, the quality and reliability of institutional guidelines used globally have been the subject of considerable discussion and evaluation [7–14]. Recommendations made by expert panels contributing to these universal guidelines require endorsement and acceptance by national physicians to facilitate their integration into routine clinical practice. In this context, the utility of society-based international guidelines has been scrutinized in several studies, which have highlighted that a substantial portion of the information within these guidelines is neither read nor implemented [15, 16].

Although the recommendations within these guidelines are derived from evidence-based data and are invaluable for designing diagnostic and therapeutic algorithms, their acceptance and practical application are often influenced by specific local factors that warrant careful consideration. Consequently, the adoption and implementation of these well-prepared and widely published guidelines may differ significantly from the intended outcomes due to these local contextual variables [15–17].

In light of these considerations, this study aims to investigate the potential impact of local factors and perceptions on the acceptance and application of the recommendations outlined in “urolithiasis guidelines” for the effective and safe management of urolithiasis.

## Materials and methods

This survey study targeted urologists across the country, with a primary focus on those involved in the management of urolithiasis, aiming to gather insights and experiences regarding the use of urolithiasis guidelines in local clinical practice. Participants were identified using contact information retrieved from professional group databases. The target population consisted of urology professionals capable of sharing their experiences and perspectives on clinical practice patterns related to the utilization of published urolithiasis guidelines.

A total of 415 urology specialists were invited to participate in the survey, which was disseminated via Google Forms. Participation was voluntary, and 270 respondents completed the survey, yielding a response rate of approximately 65.06%, which was considered sufficient for subsequent analyses.

The survey comprised 22 questions designed to evaluate the role of urolithiasis guidelines in daily clinical practice in Turkey, based on the personal approaches of experienced urologists in stone management. Responses were collected electronically through the Google Forms platform. All responses were anonymous, and personal data were kept strictly confidential. Upon completion of the survey, the responses from the 270 participants were automatically transferred to a database for statistical analysis.

The collected data were analyzed using the Statistical Package for the Social Sciences (SPSS) software. Given the nature of the survey, the anonymity of participants, and the focus on professional opinions and experiences, ethical committee approval was not sought.

## Results

A total of 415 urologists were invited to participate in the survey, and 270 responded (response rate: 65.06%). This response rate is notably higher than those reported in similar studies [10–13]. Consistent with our findings, Fadrique GG et al. also reported outcomes related to the use of local guidelines in Spain [18].

Analysis of the results revealed several key findings that illuminate the influence of urolithiasis guidelines on clinical practice patterns in Turkey. All survey participants primarily focused on the management of stone disease, with 40,9% reporting that they performed at least 20 stone-related procedures per month. The respondents’ ages ranged from 25 to 78 years (mean: 41.02 ± 12.13), while their professional experience spanned from 1 to 47 years (mean: 13.6 ± 11.7). In terms of monthly procedure volume, 30.7% of respondents performed fewer than 10 procedures, 28,5% performed between 10 and 20, and 40,9% performed more than 20.

Nearly half (48.9%) of the urologists reported receiving no formal education or training on the use of guidelines in clinical practice during their careers. Among the respondents, 56.9%—predominantly those working in universities or educational/training hospitals—reported using guidelines as part of a clinically accepted practice pattern, whereas 41.6%—mainly those working in private hospitals or clinics—used the guidelines on an individual basis.

**Fig. 1:**
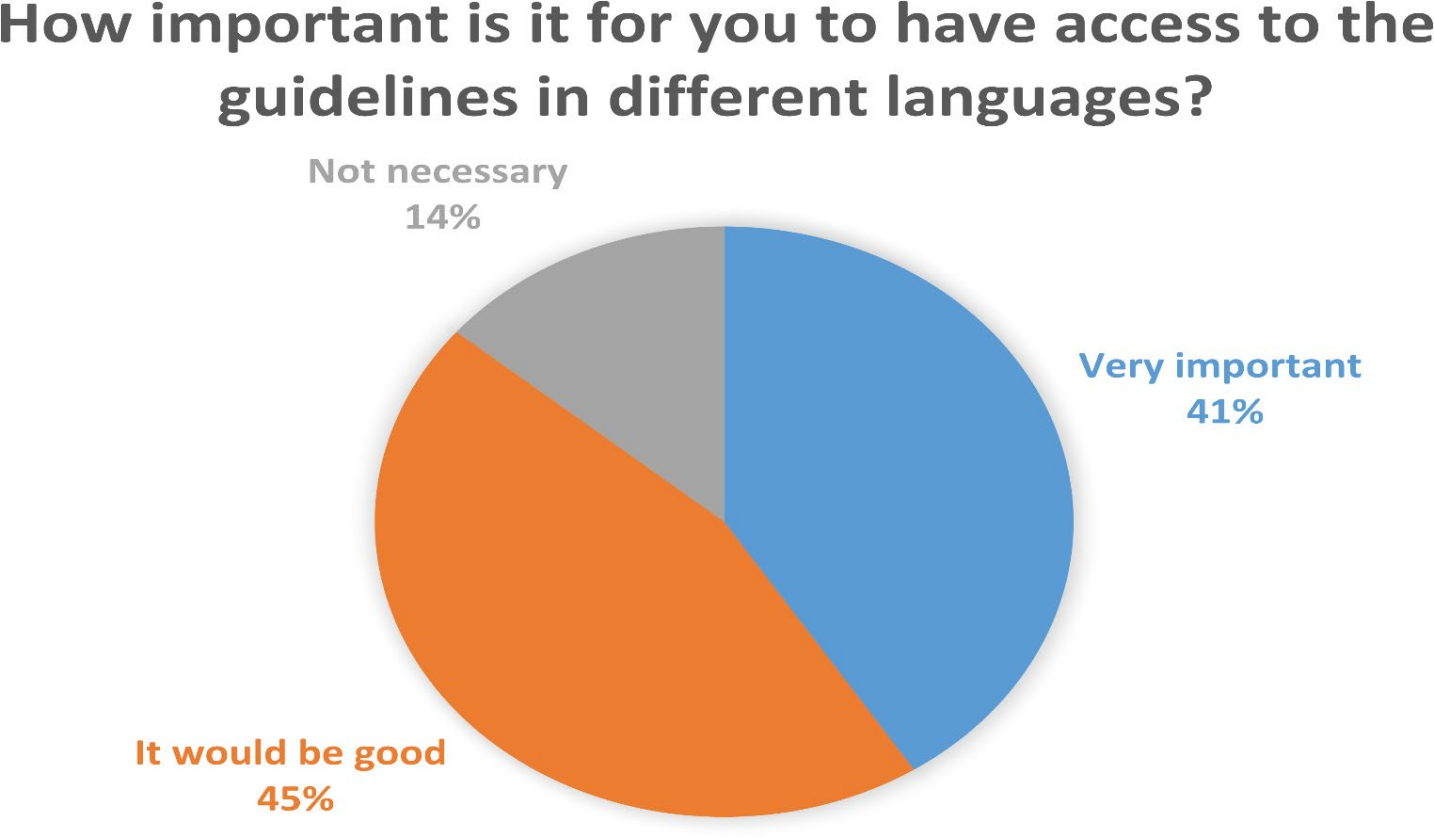
Evaluation of access to guidelines in different languages

Regarding access to and use of guidelines in different languages, 40.9% of respondents deemed access to these versions “very important,” while 45.3% indicated it would be “good to have,” and 13.9% considered them “not necessary.” (Fig. 1) Additionally, 19% reported having pocket-sized versions of the guidelines readily available, 76.6% kept them on their desks for reference, and 4.4% did not use them in clinical practice at all. Less experienced urologists and residents were more likely to use Turkish versions of the guidelines, whereas experienced practitioners reported less frequent use. Notably, 74.5% of respondents perceived no significant differences between local guidelines and those from international societies regarding content and potential benefits.

With respect to accessibility, 92.7% of urologists found the guidelines easy to access and use, while 7.3% described the process as difficult and time-consuming. Regarding sources for obtaining guideline information, 84.7% utilized relevant associations’ websites, 8.8% relied on printed booklets in their local language, 5.1% accessed original printed booklets, and 1.5% reported not using any guidelines.

When asked about the frequency of guideline use, 53.7% of urologists reported consulting them when faced with unfamiliar cases, 22.1% used them in all cases, and another 22.1% only referred to them in complex cases. A small proportion (2.1%) reported never using the guidelines in their daily practice.

**Fig. 2:**
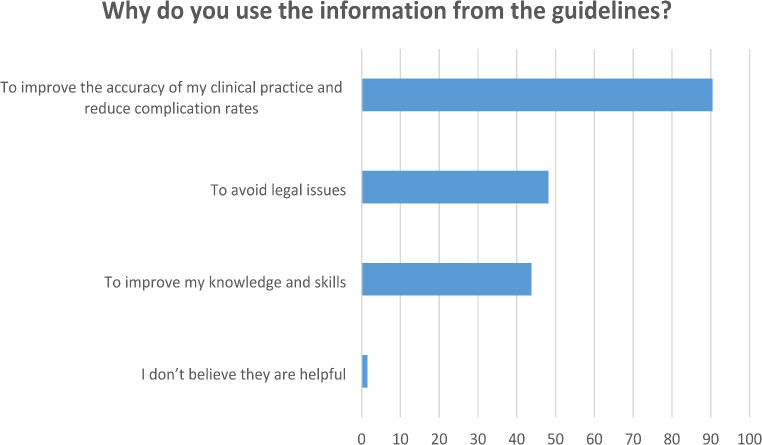
Reasons of using guidelines

The primary purpose of using guidelines was also investigated, with 90.5% of participants stating they used them to enhance the accuracy of their clinical practice and reduce complication rates. Additionally, 48.2% reported using guidelines to avoid legal issues, while 43.8% aimed to improve their clinical knowledge and skills. Only 1.5% found guidelines to be of no benefit (Fig. 2).

**Fig. 3:**
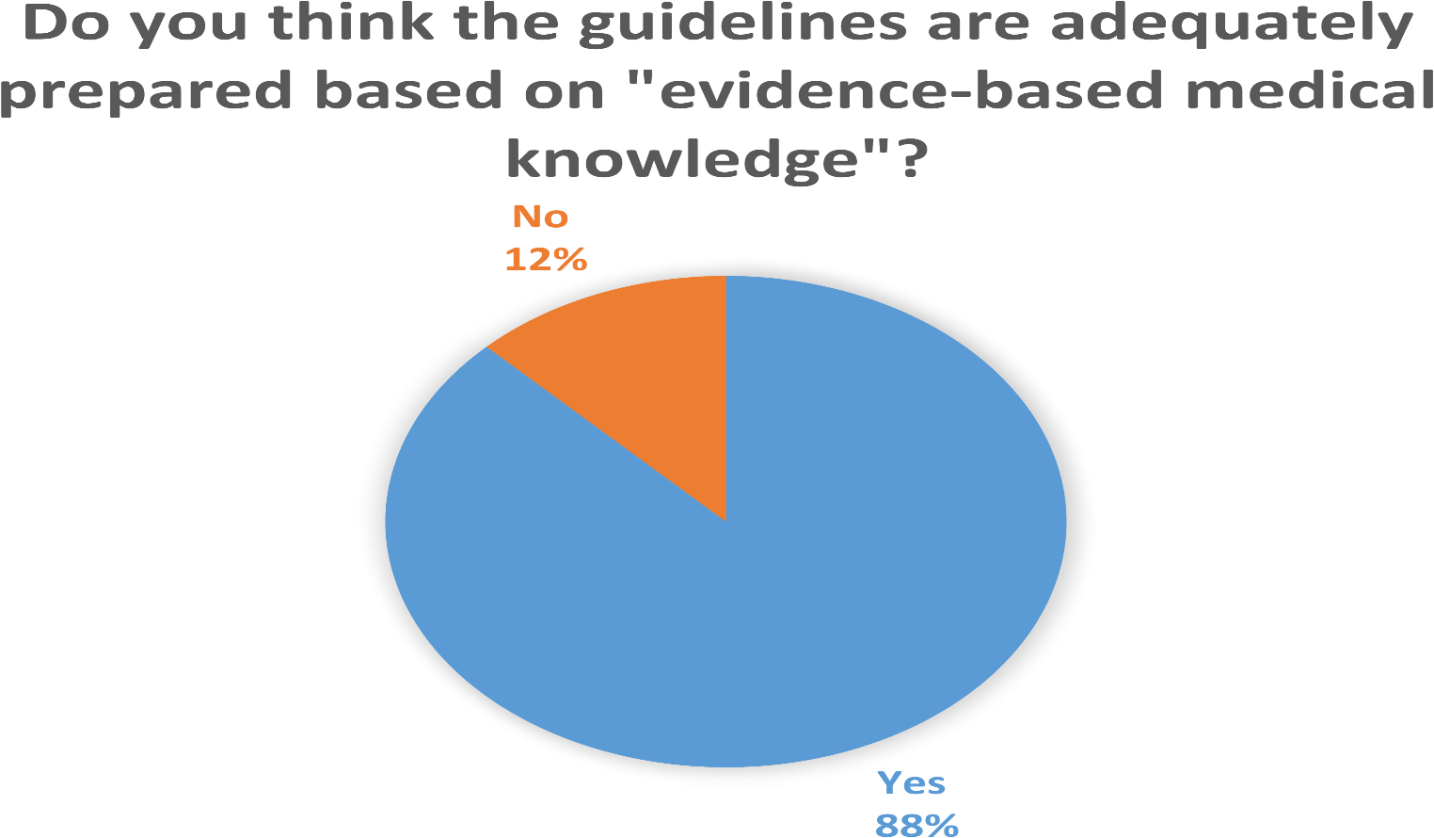
Evaluation of the opinion on whether the guidelines were prepared in accordance with evidence-based medicine

The reliability of guidelines for clinical applications was a critical issue addressed in the survey. A majority (65.7%) expressed concerns that the personal opinions and beliefs of panel members could influence the preparation and recommendations presented in guidelines. When asked if the guidelines were sufficiently evidence-based, 87.6% of participants responded affirmatively, while 12.7% believed the guidelines might lack adequate evidence to fully rely on them (Fig. 3).

Regarding guideline usage patterns, 98.5% of respondents reported using the European Association of Urology (EAU) guidelines, 41.6% used the American Urological Association (AUA) guidelines, 6.6% referred to the International Alliance of Urolithiasis (IAU) guidelines, 0.7% consulted the Asian Urological Association (Asian AU) guidelines, and 20.4% used local guidelines in their native language. Only 1.5% of respondents utilized all available guidelines in their clinical practice.

Finally, the inclusion of patients in guideline preparation was highlighted as an important consideration. A total of 65.7% of respondents emphasized the importance of patient involvement in the content development process. They believed this could provide valuable insights into patient perspectives, aiding urologists in making informed decisions during clinical management.

## Discussion

Guideline-based recommendations are widely regarded as a valuable resource for practicing urologists and residents in training, enabling evidence-based decision-making derived from rigorously conducted studies. These recommendations play a crucial role in aiding physicians during the complex decision-making process, helping them identify the most appropriate treatment options among available alternatives. The credibility of these guidelines stems from the rigorous and detailed evaluation of evidence-based data within the literature. Consequently, clinical guidelines are considered a reliable source of information, not only for healthcare providers but also for insurance companies, as they aim to enhance the quality of care delivered in clinical practice [19, 20]. 

This study aimed to evaluate local practice patterns regarding the use of urolithiasis guidelines in Turkey. To our knowledge, this is the first study to focus on critical factors influencing the clinical application of these guidelines by national urologists, particularly those involved in the management of urolithiasis.

Studies addressing the attitudes of urologists toward the local use of urolithiasis guidelines are extremely limited. The only comparable study, conducted by Fadrique GG et al., assessed adherence to clinical guidelines for urinary stone management. However, that study involved only 12 urologists from 8 hospitals, evaluating their decisions in 723 cases. It revealed lower-than-expected adherence rates to guideline recommendations, with higher compliance observed for managing smaller renal and ureteral stones, while lower adherence was noted for planning treatments for larger stones [18].

Our study provides several key findings that highlight the current state of local practice patterns regarding urolithiasis guidelines in Turkey. Most notably, 82.5% of participating urologists were highly experienced in stone management, performing at least 20 procedures per month. Among all respondents, 84.7% reported benefiting from guideline recommendations to varying degrees, while a small but notable percentage (2.1%) did not use guidelines in their routine practice.

Access to and effective use of local (Turkish) versions of the guidelines varied according to the participants’ level of experience. While less experienced urologists and residents tended to rely more heavily on these versions, experienced practitioners used them less frequently. This disparity does not necessarily imply that experienced urologists disregard guidelines but rather reflects their accumulated clinical expertise and proficiency in utilizing other resources, including international guideline versions. Importantly, 41% of respondents emphasized the utility of having local guideline texts readily available for reference.

Half of the respondents reported receiving no formal training or education on the preparation, content, or use of guidelines during their careers. This highlights the need for targeted educational efforts, particularly for medical students and residents in training. Introducing guideline-based recommendations through university and training hospital programs, as part of presentations or clinical case discussions, could familiarize future practitioners with their importance and application. For instance, guidelines could be incorporated into annual educational activities, with residents presenting recommendations to their peers and supervisors.

Despite the high level of experience among the majority of participants (82.5%), the rate of regular use of guidelines was lower than expected. This finding suggests that experienced urologists may feel sufficiently knowledgeable to apply guideline principles without consulting the texts directly. While almost all respondents reported using European Association of Urology (EAU) guidelines, only about half utilized American Urological Association (AUA) guidelines, reflecting the geographical and professional context of the participants practicing in a European region.

The primary motivations for using guideline-based information in clinical practice were improving treatment standards (90.5%) and reducing complication risks. Additionally, 48.2% of respondents used guidelines to mitigate legal risks, while 43.3% sought to enhance their knowledge and skills. Interestingly, nearly half of the participants viewed guideline recommendations as beneficial for minimizing legal risks, a concern that is gaining increasing relevance in contemporary medical practice. This underscores the potential role of guideline recommendations in legal evaluations of complex cases with severe complications.

Another critical finding was the perception among many respondents that the personal opinions and beliefs of guideline committee members could influence the content and reliability of recommendations. Supporting this, 12.7% of participants expressed skepticism regarding the evidence-based foundation of guideline recommendations, suggesting that some guidelines may lack sufficient rigor in their preparation. These insights indicate a need for guideline committees to adopt new approaches that enhance transparency, reliability, and user confidence.

Our study is not without limitations. First, the survey included many questions, and the time-consuming nature of the questionnaire may have contributed to a lower response rate. Although a follow-up email was sent one month after the initial invitation, participation remained lower than anticipated. Additional reminder emails might have improved the response rate, but logistical constraints prevented this. Nonetheless, as the first study examining the local application of urolithiasis guidelines, we believe our findings provide valuable insights into the approaches of Turkish urologists to these recommendations.

## Conclusions

The available guidelines for the management of urinary stones are widely regarded as valuable resources, offering essential recommendations to ensure successful outcomes for various treatment modalities in clinical practice. However, findings from this survey study highlight that clinical practice patterns can vary significantly under local conditions, influenced by the experience and attitudes of local urologists toward these guidelines. Furthermore, the application of guideline recommendations may differ across countries, depending on perceptions regarding their preparation, content, and practical applicability. We believe that insights gathered from local physicians can contribute to a deeper understanding and more rational use of these guidelines, ultimately improving their interpretation and clinical utility.

## Electronic supplementary material

Below is the link to the electronic supplementary material.

Supplementary Material 1

## Data Availability

No datasets were generated or analysed during the current study.
