# Supplementary material for: Local clinical practice patterns in urolithiasis guidelines: a critical evaluation from Turkey
Source: World J Urol. 2025 Feb 3;43(1):97. doi: 10.1007/s00345-025-05490-y (PMC11790800; doi:10.1007/s00345-025-05490-y)
Supplement: Supplementary file 1 — Supplementary Material 1 [file 345_2025_5490_MOESM1_ESM.pdf]

Urolithiasis Guidelines Awareness and Accessibility Survey

1. How old are you?

2. What is the name of the clinic you work at?

3. How many years of experience do you have in the field of urology?

4. What is the average number of stone surgeries/procedures you perform per month?

- <10
- 10-20
- 20

5. How many stone patients do you typically encounter in a day?

- <5
- 5-10
- 10

6. How important is it for you to have access to the guidelines in different languages?

- a. Very important
- b. It would better
- c. Not important

7. Do you receive any training or guidance to use the guidelines in your clinical practice?

- a. Yes
- b. No

8. Are there significant differences between the guidelines in your country (health regulations, hospital regulations) and international guidelines?

- a. Yes
- b. No

9. Do you carry a stone disease guideline (pocket guide) with you at all times?

- A Yes, always
- b. No
- c. I don’t use

10. Do you think the information on stone disease in the current guidelines provides practical insights for clinical practice?

- a. Yes, it is very practical and I benefit greatly from it
- b. No, it is not practical and mostly presents general textbook information; it should be more case- and clinically oriented

11. Do you have knowledge of how the information in the guidelines is prepared?

- a. Yes
- b. No

12. Can you easily access the guidelines?

- a. Yes
- b. No

13. How do you obtain the guidelines?

- a. By visiting the websites where the guidelines are hosted
- b. Through printed original guidelines
- c. Through printed guidelines in the local language
- d. I don't use them

14. How frequently do you utilize and apply the guidelines in your clinical practice?

- a. In every case
- b. In cases where I don't have enough information
- c. In difficult cases
- d. I don't use them

15. How applicable are the recommendations in the guidelines considering the conditions in your country?

- a. Very high, applicable in every case
- b. Limited, applicable only in certain cases
- c. Not applicable at all

16. For what purposes do you use the information from the guidelines? (Multiple answers possible)

- a. To improve the accuracy of my clinical practice and reduce complication rates
- b. To avoid legal issues
- c. To improve my knowledge and skills
- d. I don't believe they are helpful

17. Do you think the guidelines adequately include the patients' perspectives and approaches?

- a. Yes
- b. No

18. Do you think it is possible that personal opinions of the guideline developers may influence the guidelines and recommendations?

- a. Yes
- b. No

19. Do you think the guidelines are adequately prepared based on "evidence-based medical knowledge"?

- a. Yes, I believe so
- b. No, I don't think so

20. Which of the following guidelines have you had the opportunity to use and apply?

- a. European Association of Urology (EAU) guidelines
- b. American Urological Association (AUA) guidelines
- c. International Alliance of Urolithiasis (IAU) guidelines
- d. Asian Association of Urology (AAU) guidelines
- e. National guidelines in local language
- f. All of the above
- g. None of the above

21. Do you apply the information from the current guidelines clinically as part of a protocol or individually?

- a. As part of a protocol
- b. Individually
- c. I do not apply them

22. What do you think should be done to make the current guidelines more practically applicable in clinical practice, and to increase their implementation rate and reliability?
